# MiR-9 and the Midbrain-Hindbrain Boundary: A Showcase for the Limited Functional Conservation and Regulatory Complexity of MicroRNAs

**DOI:** 10.3389/fcell.2020.586158

**Published:** 2020-11-23

**Authors:** A. Alwin Prem Anand, Gonzalo Alvarez-Bolado, Andrea Wizenmann

**Affiliations:** ^1^Institute of Clinical Anatomy and Cell Analysis, University of Tuebingen, Tuebingen, Germany; ^2^Department of Neuroanatomy, University of Heidelberg, Heidelberg, Germany

**Keywords:** neural development, mid-hindbrain boundary, danio, gallus, xenopus, Fgf8, miR-9, hairy-1

## Abstract

MicroRNAs regulate gene expression at post-transcriptional levels. Some of them appear to regulate brain development and are involved in neurodevelopmental disorders. This has led to the suggestion that the role of microRNAs in neuronal development and function may be more central than previously appreciated. Here, we review the data about miR-9 function to depict the subtlety, complexity, flexibility and limited functional conservation of this essential developmental regulatory system. On this basis we propose that species-specific actions of miR-9 could underlie to a large degree species differences in brain size, shape and function.

## Introduction

MicroRNAs (miRNAs, miRs) are short non-coding RNA oligonucleotides (18–25 bases), which regulate gene expression at post-transcriptional levels by binding specific mRNAs and in this way marking them for enzymatic destruction (reviewed in Pasquinelli, 2012). The downregulation of a gene by a miR can result in a reciprocal negative feedback signaling between the specific miR and its target gene(s). It also can form a positive feedback loop when the miR reduces expression of another miR or of an inhibitory factor (Avraham and Yarden, 2012). One miR can theoretically regulate hundreds of target genes because the miR sequence never matches completely the target mRNA sequence. For this reason, target verification for any given miR is a challenge. Several miRs seem to work as fine-tuning regulators of brain development since they reinforce or disrupt developmental or transitional stages (reviewed in Coolen and Bally-Cuif, 2009; Petri et al., 2014; Davis et al., 2015; Rajman and Schratt, 2017). Some of those have been associated with neurodevelopmental disorders such as Autism Spectrum Disorder, Down syndrome, Rett syndrome and schizophrenia (reviewed in Im and Kenny, 2012; Banerjee-Basu et al., 2014; Sun and Shi, 2015). This has led to the suggestion that their role in neuronal development and function may be more central than previously appreciated (Davis et al., 2015). In this mini-review, we will discuss miR-9 actions in the neural tube with a specific focus on the mid-hindbrain-area. We hope to show how complex and flexible the functional conservation of this essential developmental regulatory system is. Our analysis points to a species-specific set of miR-9 interactions, which (1) could crucially hone some aspects of development in some CNS regions; and (2) could therefore achieve species differences in brain size, shape and function.

MiR-9 is conserved from flies to humans (Yuva-Aydemir et al., 2011) and primarily expressed in the central nervous system (CNS) at least in vertebrates (Wienholds et al., 2005; Deo et al., 2006; Kloosterman et al., 2006; Kapsimali et al., 2007; Radhakrishnan and Alwin Prem Anand, 2016). However, the extent of its functional conservation is not known. Studies on the CNS of different species and on neural stem cells (NSCs) have disclosed an important role of miR-9 in balancing proliferation and differentiation of neural progenitor cells (NPCs) and NSCs (Leucht et al., 2008; Packer et al., 2008; Shibata et al., 2008, 2011; Yoo et al., 2009; Zhao et al., 2009; Bonev et al., 2011; Roese-Koerner et al., 2017). MiR-9 knockout (KO) as well as overexpression (OE) experiments result in smaller or larger brains or specific brain regions and confirm the role of miR-9 in neural proliferation-differentiation balance (reviewed in Coolen et al., 2013). MiR-9 is also important to keep the quiescence/activation balance of adult NSCs in zebrafish telencephalon (Katz et al., 2016) and in human and mouse adult NSCs (Zhao et al., 2009; Roese-Koerner et al., 2017). Moreover, miR-9 expression is also upregulated after Zika virus infections in the developing mouse telencephalon (Zhang et al., 2019).

MiR-9 expression is tightly regulated by several genes and by other miRs (Packer et al., 2008; Denli et al., 2009; Bonev et al., 2012; Coolen et al., 2013; Davila et al., 2014). MiR-9 is repressed by the neurogenic repressor REST and its cofactors (SCP1, CoREST) as well as by TLX in mouse cortex (Packer et al., 2008; Zhao et al., 2013). The Notch effector HES1/HAIRY1 also regulates miR-9 in mouse cortex (Bonev et al., 2012; Tan et al., 2012). *Ngn1* inhibits astrogliogenesis through induction of miR-9 (Zhao et al., 2015). And ElAV2 counteracts the suppression of miR-9 by binding to U-rich region of *Foxg1* mRNA (Shibata et al., 2011). The all trans retinoic acid and retinoic acid have been shown to induce miR-9 (Kutty et al., 2010).

MiR-9, in turn, regulates a variety of genes to balance proliferation and differentiation in telencephalon, hindbrain, spinal cord, and (*in vitro*) in NSCs (reviewed in Coolen et al., 2013). It induces the switch of BAF5a to BAF53b, an epigenetic regulator (Yoo et al., 2011; Tang et al., 2013). Other reported targets of miR-9 include *Foxg1*, *Foxp2*, *Gsh2*, *SIRT1*, and *REST* (Shibata et al., 2008, 2011; Clovis et al., 2012). In the developing mouse cortex, miR-9 targets Foxg1, Nr2e1, Gsh2, and Meis2 (Shibata et al., 2011).

Mir-9 reinforces it’s own expression by targeting *REST*, *TLX*, and *HES1* in forming auto-regulatory loops (Bonev et al., 2012; Tan et al., 2012; Goodfellow et al., 2014; Roese-Koerner et al., 2017). In spinal cord, *FOXP1* (Otaegi et al., 2011) and OC1 (onecut transcription factor) (Luxenhofer et al., 2014) were reported as miR-9 target genes.

In particular, *Hes* genes are a recurring target of miR-9 in forebrain and in NSCs, and the intensity of their expression oscillates with that of miR-9 to balance neurogenesis and proliferation (Bonev et al., 2012; Tan et al., 2012; Goodfellow et al., 2014; Roese-Koerner et al., 2017). Across vertebrates, *Hes1/her6* genes have a conserved 3′UTR binding site for miR-9. MiR-9 targeting of her/hairy/hes is necessary to properly balance progenitor proliferation genes in zebrafish, *Xenopus* and mouse (Leucht et al., 2008; Bonev et al., 2011, 2012; Coolen et al., 2012). In all three model animals, miR-9 and *Hes1* form a regulatory loop. This loop is also active in human neural stem cells (Roese-Koerner et al., 2017) and helps to steady the ultradian Hes oscillation (Kageyama et al., 2008), necessary for controlled neural proliferation.

Particularly interesting is the fact that miR-9 can regulate different target pathways in forebrain and hindbrain, to obtain region-specific results (Bonev et al., 2011).

## miR-9 Function at the Midbrain-Hindbrain Region

The function of miR-9 in the development of the CNS has been approached by gain of function (Gof) using oligonucleotide mimics or plasmid vector OE and loss of function (LoF) by KO or anti-miRs experiments in different brain regions and in the spinal cord of animal models from different vertebrate classes. The results show that miR-9 is essential for proper neural differentiation but that its effect is not uniform in all vertebrate models and cannot be easily generalized. As an example, LoF and OE in mouse and *Xenopus* forebrain suggest that miR-9 is necessary for the production of the early population of neurons (Shibata et al., 2008, 2011; Bonev et al., 2011; Shu et al., 2019a,b). More specifically, in the cortex, miR-9 is essential for the specification of the first-born cortical layers (Shu et al., 2019b). On the contrary, miR-9 is necessary for the differentiation of late born motor neurons of the spinal cord (Otaegi et al., 2011; Luxenhofer et al., 2014).

Since the variety of results on different CNS regions of different models makes it difficult to generalize, here we would like to focus on the midbrain-hindbrain region (MHB). The role of miR-9 in the development of the MHB showcases the major themes of complexity, subtlety and species-specificity.

**FIGURE 1:**
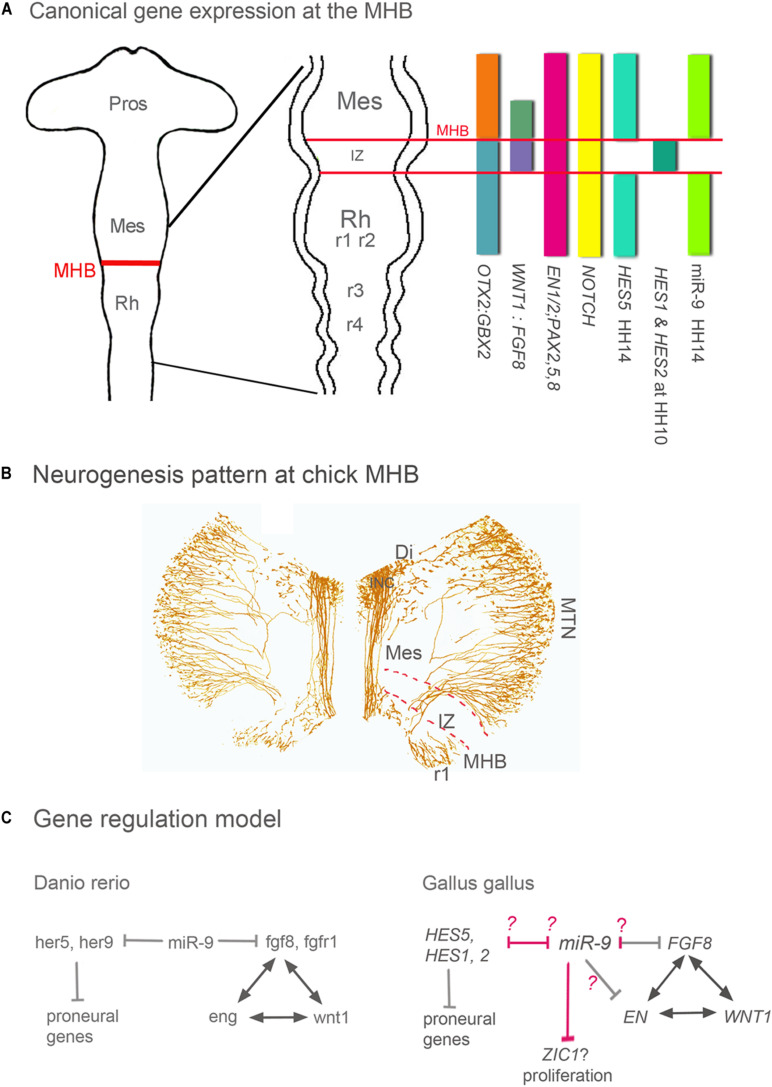
Gene expression, neurogenesis and gene regulation around the MHB. **(A)** Summary of gene expression patterns around the MHB. *Otx2*, *Gbx2*, *Wnt1*, *Fgf8*, *En1/2*, *Pax2* -*5* -*8*, and *Notch* are expressed in a similar pattern in all vertebrates. The expression pattern of Hes genes however differs between vertebrate species. For instance, the pattern of zebrafish her 9 is similar to that of chick HES5. **(B)** Pattern of neurogenesis around the chick MHB at HH17 (∼E3). Differentiated neurons were labeled with an antibody against medium weight neurofilament (RMO-270). The IZ lacks neurons at this stage, and in midbrain only the dorsally located MTN neurons have developed. ICN neurons are located in ventral diencephalon and form the medial longitudinal tract left and right of the FP. In r1 ventral and dorsal neurons have differentiated. **(C)** In all vertebrates studied, during the maintenance phase, *Wnt1*, *Fgf8*, *Pax*2/5/8, and *En* genes regulate each other to maintain the MHB. In zebrafish and chick, miR-9 suppresses *Fgf8* expression and thus indirectly the expression of *Wnt1*, *Pax*, and *En* genes. The difference between both species is in the miR-9 targeting. In chick, *Fgf8*, and *En1* are target genes of miR-9; where the former shows experimental reduction and the later is inconsistent (Alwin Prem Anand et al., 2018). In zebrafish, miR-9 promotes neurogenesis by inhibiting different Hes genes around and within the IZ (her9 and her5, respectively). In chick, none of the Hes genes expressed in and around the IZ are miR-9 targets (pink arrows). miR-9 OE in chick resulted in immature neurogenesis only in r1 and IZ but not in midbrain, These results suggests that at least in chick IZ and in hindbrain genes of the Hes pathway could be inhibited by miR-9. In chick midbrain, like in *Xenopus* telencephalon (Bonev et al., 2011) growth is reduced but without an effect on cell death as in *Xenopus*, which might be mediate by inhibiting the Wnt activator Zic1. The Models are modified from Rhinn and Brand (2001) and Delaloy and Gao (2008). Di, diencephalon; FP, floor plate; INC, interstitial nucleus of Cajal; IZ, intervening zone; MES, mesencephalon; MHB, mid-hindbrain boundary; MTN, mesencephalic trigeminal nucleus; Pros, prosencephalon; Rh, rhombencephalon; r1 to 4, rhombomeres 1–4.

The Intervening Zone (IZ) is a region rostral to the MHB and separates midbrain from hindbrain (Palmgren, 1921; Vaage, 1969; Bally-Cuif and Wassef, 1994; Wullimann and Knipp, 2000). The IZ expresses Fgf8, undergoes neurogenesis later than neighboring areas and is crucial for MHB maintenance and MH development (Figures 1A,B; Palmgren, 1921; Vaage, 1969; Bally-Cuif and Wassef, 1994; Wullimann and Knipp, 2000). The IZ does not express miR-9 in zebrafish, *Xenopus* and chick (Leucht et al., 2008; Bonev et al., 2011; Figure 1A). Such a miR-9-free zone has not been explicitly described in the mouse, but it is visible e.g., in the expression patterns published by Shibata et al. (2008). The formation and maintenance of the IZ region in zebrafish are based on active inhibition of neurogenesis and expression of the hairy/E(spl) gene her5 (Geling et al., 2003). Her5 together with the her-like gene “him” suppresses neurogenesis and sustains in this way the growth of the entire mid-hindbrain area (Tallafuss and Bally-Cuif, 2003). MiR-9 OE experiments cause premature neurogenesis in the IZ and rostral hindbrain in zebrafish (Leucht et al., 2008) and chick (Alwin Prem Anand et al., 2018). Interestingly, in chick and *Xenopus* Hairy1/Hes1 is not expressed in the IZ, although it shows a predicted miR-9 binding site in the 3′UTR (Bonev et al., 2011; Alwin Prem Anand et al., 2018). In chick, *FGF8* and *EN1* are target genes of miR-9, where the former shows consistent experimental reduction, the latter does not (Alwin Prem Anand et al., 2018).

In zebrafish, miR-9 suppresses not only her5 but also genes of the fgf pathway (fgf8, fgfr1, and canopy 1; Figure 1C) at the MHB, thus affecting positioning, establishment, and maintenance of the MHB. Indeed, in zebrafish, miR-9 overexpression can completely abolish fgf8 expression in the MHB and thus the development of the early MHB (Leucht et al., 2008). Chick and zebrafish show miR-9 target binding sites for *Fgf8/fgf8* (Leucht et al., 2008; Alwin Prem Anand et al., 2018). The following example is particularly interesting since it reveals a regional specificity in the mechanisms of miR-9 function that depends on regionally expressed downstream genes. In *Xenopus*, the function of miR-9 promoting neurogenesis and antagonizing proliferation is mediated by decreasing the availability of Hairy1 but while in the forebrain the final effect of this decrease is mediated by Fgf8, in the hindbrain it is mediated by Wnt (Bonev et al., 2011).

In chick, miR-9 OE or LoF resulted in either a smaller or a larger zone of *FGF8* expression at the MHB, respectively, but never in a complete loss of the MHB domain and FGF8 expression like in zebrafish (Leucht et al., 2008). Thus, after miR-9 OE (Alwin Prem Anand et al., 2018), the chick MHB continued to express not only FGF8, but also typical regional markers *WNT1*, *EN1*, and *EN2* (Rhinn and Brand, 2001; Wurst and Bally-Cuif, 2001; Raible and Brand, 2004; Dworkin et al., 2012). Nevertheless, FGF8 expression was affected, as was indirectly WNT expression (Alwin Prem Anand et al., 2018). Thus, one of the tasks of miR-9 in chick seems to restrict FGF-8 expression (Figure 1C) and thus the extent of the IZ. Since the size of the IZ is important for the growth and patterning of the MH area, miR-9 influences the size of that area. Several HES genes are expressed in and around the chick IZ (Figure 1A). So far none comparable to her5 in zebrafish and Her2 in mouse (Leimeister et al., 1999; Tallafuss et al., 2003) has been described. *HAIRY1/HES1* and *HAIRY2*/HES2 are only temporarily expressed in the IZ in chick (Tossell et al., 2011). HES1 seems to be the ortholog of her9 in zebrafish (Leve et al., 2001); however, its expression pattern correlates only transiently with that of her5 in zebrafish. Chick *HES5* on the other hand is expressed along the entire MH area except for the IZ and correlates rather with the expression pattern of zebrafish her9 (Figure 1A; Kimura et al., 2004). Alas, so far miR-9 has shown no theoretical target-binding site for chick HES2 or HES5, and in chick we have not observed downregulation of *HAIRY1/HES1* expression after miR-9 OE although there is a theoretical target site (Alwin Prem Anand et al., 2018). Nevertheless, miR-9 OE causes premature neurogenesis in posterior MHB, i.e., in the IZ and in anterior hindbrain of the chick (Alwin Prem Anand et al., 2018), as reported in zebrafish MHB (Leucht et al., 2008) and in anterior hindbrain of Xenopus (Bonev et al., 2011). In addition, or instead of HES genes their target NOTCH might be downregulated by miR-9. NOTCH is known to block miR-9 expression in neural stem cells (Roese-Koerner et al., 2016). This suggests that the NOTCH-HES pathway in chick is interrupted and proneural genes are activated.

## Discussion

Although miR-9 has an effect on Fgf8 expression in the MHB both in chick and zebrafish, the consequences of over-expression are never as severe in chick as in zebrafish. This could be an artifact of timing and targeting of the experiments in chick, which were performed unilaterally and only after the MHB was formed (Alvarado-Mallart et al., 1990; Itasaki et al., 1991). In the experiments, miR-9 OE correlated approximately with the beginning of activity of miR-9 around the MHB in chick at Embryonic day (E) 1.5 (or HH 14 (Hamburger and Hamilton, 1951; Alwin Prem Anand et al., 2018; Figure 1A). In contrast, in the zebrafish experiments, miR-9 manipulations were performed in oocytes, long before the MHB is positioned and formed and before miR-9 is expressed in the MH area (Leucht et al., 2008). This early overexpression may explain the more profound effects in zebrafish. These results suggest that an early KO of miR-9 might have additional effects on neural tube development and thus influence MHB development.

There is another interesting difference between species. Although miR-9 overexpression causes premature neurogenesis in the IZ in zebrafish and chick embryo and in anterior hindbrain in zebrafish, chick and *Xenopus* (Leucht et al., 2008; Bonev et al., 2011; Alwin Prem Anand et al., 2018), neither chick nor *Xenopus* seem to express Hes1/Hairy1 in the IZ. In the *Xenopus* hindbrain (Bonev et al., 2011) and possibly also in the chick MHB (Alwin Prem Anand et al., 2018), miR-9 targets *zic1*, a Wnt activator (Figure 1C), in this way reducing proliferation, a step previous to neurogenesis induction. Is miR-9 then an inductor of neurogenesis? Results obtained in the chick midbrain, immediately rostral to the MHB, where broad ectopic miR-9 OE results in reduced proliferation but no ectopic neurogenesis (Alwin Prem Anand et al., 2018), suggest the opposite. Alternatively, the chick midbrain could have a very strong antagonist to miR-9 to inhibit premature neurogenesis. It will be interesting to see if this is also the case in other species.

Our synopsis of all these results from different species is that miR-9 has essential, complex and time-dependent but only partially conserved functions in vertebrates. These make this intricate system difficult to approach. At the same time, the identification of species-specific regulation of miR-9 expression is yielding new insights on the different mechanisms that regulate the spatiotemporal functions of miR-9. One conclusion that can be drawn is that the miR system has evolved to be flexible, species-specific, subtle and time dependent. On the basis of this mini-review we tentatively propose that (1) maybe there are species-specific sets of miRs governing certain aspects of development; (2) this could be part of the cause of the differences in brain size, shape, and function in different vertebrate classes.

## Author Contributions

AAPA, GA-B, and AW wrote the manuscript. AAPA and AW designed the figure. All authors contributed to the article and approved the submitted version.

## Conflict of Interest

The authors declare that the research was conducted in the absence of any commercial or financial relationships that could be construed as a potential conflict of interest.
